# Outcomes Post-Nick's Aortic Root Enlargement Technique: Single-Center Experience

**DOI:** 10.1055/s-0042-1757800

**Published:** 2022-12-20

**Authors:** Amr Ashry, Sashini Iddawela, Vaibhav Mishra, William Wang, Heba M. Mohammed, Amer Harky, Mohammed M. Mostafa

**Affiliations:** 1 Department of Cardiothoracic Surgery, Assiut University Hospital, Faculty of Medicine, Assiut University, Assiut, Egypt; 2 Department of Paediatric Cardiac Surgery, Alder Hey Children Hospital, Liverpool, United Kingdom; 3 Department of General Surgery, University Hospitals Birmingham, United Kingdom; 4 St George's Medical School, University of London, Cranmer Terrace, London, United Kingdom; 5 Barts and The London School of Medicine and Dentistry, Queen Mary University of London, United Kingdom; 6 Department of Public Health and Community Medicine, Faculty of Medicine, Assiut University, Assiut, Egypt; 7 Department of Cardiothoracic Surgery, Liverpool Heart and Chest Hospital, Liverpool, United Kingdom

**Keywords:** aorta, root enlargement, technique, Nick, outcomes

## Abstract

**Background** Different techniques for aortic root enlargement (ARE) have been reported in the literature. Each technique comes with its own advantages and disadvantages. We report our outcomes of Nick's technique for ARE.

**Methods** A single-center retrospective data analysis of 31 patients was performed. Patients were operated between May 2015 and November 2017 at Assuit University Heart Hospital, Assuit, Egypt.

**Results** The median cardiopulmonary bypass time was 125 minutes (range: 90.0–160.0 minutes), with 90 minutes of cross-clamp (range: 60.0–110.0 minutes). Altogether 59% of the patients had mixed aortic valve diseases. Median intensive care unit and total hospital stay were 2 and 5 days, respectively. Patient-prosthesis mismatch was reported in one patient only (3.25%). Two patients died within 30 days. Median pressure gradient across the aortic valve was 20 mm Hg at 3 years of follow-up.

**Conclusion** The benefits of Nick's technique for ARE can be demonstrated in populations with younger patients and complicated pathology. Further research is required in larger patient populations.

## Introduction

Aortic valve replacement (AVR) is a common procedure performed in the setting of valvular diseases such as severe high-grade aortic stenosis and aortic regurgitation. A small aortic annulus can complicate the procedure and result in prosthesis-patient mismatch (PPM). PPM is an unfortunate circumstance in which the prosthesis used for AVR is too small with respect to the patient's body habitus, resulting in derangements in transvalvular pressure gradients. 1 PPM is associated with higher perioperative and long-term mortality as a result of substandard hemodynamics, pulmonary hypertension, and poor regression of left ventricular hypertrophy. 1 2

Surgical aortic root enlargement (ARE) during AVR allows for the placement of a larger size aortic valve prosthesis, reducing the risks of PPM and improving patient outcomes. 3 A posterior ARE was first suggested by Nick et al in 1970. He described extending the aortotomy posteriorly through the noncoronary sinus and then across the aortic annulus up to the origin of the mitral valve, inserting a pericardial patch to expand the annulus. 3 4

Although this method allows for a lesser degree of annular enlargement, it is simpler to perform than other ARE techniques and is less radical. 4

There is an ongoing debate around whether ARE carries additional operative risks, in particular via prolonged bypass and ischemia times, subannular bleeding, paraprosthetic leakage, suture line disruption, patch dislodgement, and aneurysm formation. 5 However, multiple studies have evidenced no increases in surgical risk or adverse events and have highlighted ARE to be a safe addition overall to AVR. 6 7

This article demonstrates a single-center experience using Nick's technique as an adjunct to AVR to reduce the risks of PPM and improve postoperative outcomes.

## Materials and Methods

Preoperative, in-hospital, and follow-up data were collected for patients requiring Nick's technique between May 2015 and November 2017 at Assuit University Heart Hospital, Assuit, Egypt. This study was approved by the Healthpoint Research Ethics committee of Assiut University, Faculty of Medicine. It was conducted according to the principles of the Declaration of Helsinki. A written informed consent was obtained from all participants. Patient demographics included age, sex, weight, height, body surface area (Mosteller formula), body mass index (BMI), past medical history of diabetes mellitus, hypertension, and atrial fibrillation (AF). The mean gradient across the stenotic aortic valve was recorded prior to surgical intervention. Intraoperative data on bypass time, ischemic time, and number of valve replacements (single or double), and which valves were involved were collected. The postoperative outcomes evaluated included length of intensive care unit (ICU) stay, length of hospitalization, duration of mechanical ventilation, reexploration for bleeding, size of aortic prosthesis, PPM, mortality, postoperative temporary pacemaker placement, and the mean gradient across the stenotic aortic valve at 3 years following surgical intervention.

### Operative Technique

Nick's root enlargement technique has been elaborated in previous literature—the primary goal is to enlarge the aortic valve annulus to avoid PPM. 8 The posterior aortic root and annulus enlargement can be performed by aortotomy extension through the noncoronary sinus across the aortic annulus. 1

Necessary investigations, such as palpation and possibly an epiaortic ultrasound, are being performed to locate healthy aortic regions for cannulation and cross-clamp placement. Routine cardiopulmonary bypass is performed with an aortic cannula placed in the distal ascending aorta and a venous drainage cannula placed in the right atrial appendage. Cardioplegia cannulas are placed for both retrograde and anterograde delivery.

An anterior transverse aortotomy is performed with the incision later being extended obliquely toward the noncoronary sinus. The valve leaflets are resected, and annular calcium deposits are removed. The aortotomy is then continued into the fibrous subaortic curtain. A patch of autologous pericardium fixed with glutaraldehyde is then applied to reconstruct the aortic defect using a 4–0 polypropylene suture which starts off the apex of the aortotomy, extends on both sides of the defect, and normally finishes approximately 2 cm beyond the native annulus. Once this is finished, a valve sizer for the anticipated valve is placed in the annulus to ensure appropriate valvular size selection and position. The valve sutures are taken in the residual part of the valve leaflets by Ethibond 2–0 interrupted sutures on Teflon pledges in a noneverting manner.

**Fig. 1:**
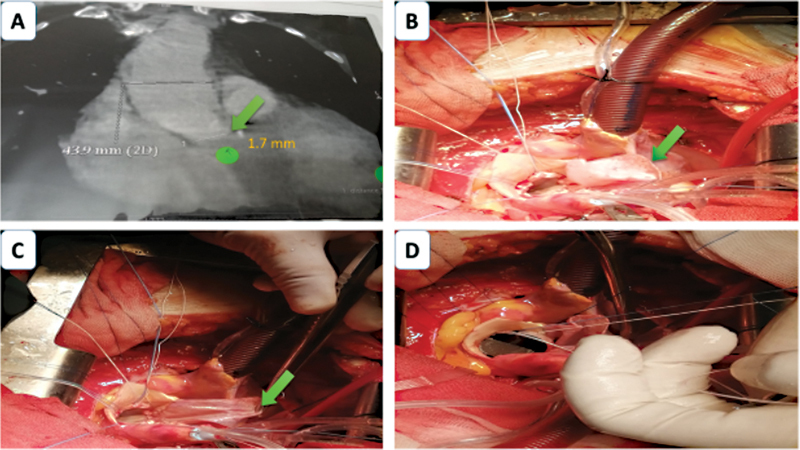
( **A)** Multislice computed tomography with aortography measuring the aortic annulus (green arrow and tag) = 1.7 mm. ( **B)** Autologous pericardium fixed with glutaraldehyde is then applied to reconstruct the aortic defect (green arrow) using a 4–0 polypropylene suture (two layers) which starts off the apex of the aortotomy. **(C)** Starting the interrupted valve sutures in the residual part of the valve leaflets by Ethibond 2–0 interrupted sutures on Teflon pledgtes in a noneverting manner. ( **D)** appropriate valve seizing and the sutures are tied. Here, in our case, we succeed to implant St. Jude reagent valve 21 mm after enlargement.

After tying the valve sutures, a careful examination of the annulus is performed to ensure proper seating of the annulus and clear visualization of coronary ostia to confirm no obstructions. Another 4–0 polypropylene suture is initiated at the aortotomy and runs continuously toward the middle as shown in Fig. 1 . The patch size is adjusted to the size of the remaining defect, and the original sutures are continued around the patch to complete the aortotomy closure. 8

### Postoperative Anticoagulation

We start early subcutaneous low-molecular-weight heparin (LMWH) bridging, as soon as the risk of postoperative bleeding is considered acceptable, may be on the second postoperative day. And we start oral VKA therapy (Warfarin) on the first or second postoperative day till the INR reach the therapeutic range, and we stop the LMWH.

### Statistical Analysis

Data were analyzed using the Statistical Package for Social Science, version 26.0 for Windows. Median and interquartile range were used to express quantitative data, while qualitative data were presented by frequencies and percentages.

## Results

Thirty-one patients underwent Nick's procedure at a single center between May 2015 and November 2017 at Assuit University Heart Hospital, Assuit, Egypt.

Surgery was performed by multiple surgeons. Patients were followed up for a median period of 38 months and echocardiographic measurements of the prosthesis were taken at this point.

### Patient Demographics and Operative Data

A summary of patient demographics and preoperative state is depicted in Table 1 . Mean age was 25 years, and gender was equally divided between males and females. Preoperative mean aortic valve gradient was 73 months. Notable comorbidities included hypertension and AF. A significant proportion had mixed aortic stenosis and regurgitation, rheumatic in origin. Eighteen patients (58.1%) received a single valve replacement. Of those who received double valve replacement, the mitral valve was also replaced. All patients had mechanical prostheses inserted, and the 19 St. Jude supra-annular valve was the most commonly used. The median aortic annulus preimplantation was noted to be 20 cm. Operative data are presented in Table 2 .

**Table 1:** Summary characteristics of included patients

Characteristic	Included patients ( *n* = 31)	
Age (y)	25.0 (22.0–34.0)	
Male sex, *n* (%)	16	51.6%
Weight (kg)	65.0 (60.0–75.0)	
Height (cm)	165.0 (165.0–170.0)	
BMI (kg/m ^2^ )	24.22 (22.03–27.42)	
Diabetes mellitus, *n* (%)	1	3.2%
BSA (m ^2^ )	1.70 (1.60–1.80)	
Hypertension, *n* (%)	3	9.7%
Controlled atrial fibrillation, *n* (%)	5	16.1%
*Aortic pathology* :		
•Mixed AS/AR, *n* (%)	19	59.3%
•Rheumatic AS only, *n* (%)	10	31.3%
•Bicuspid AS	2	6.3%
Aortic annulus (cm)	20.0 (19.0–21.0)	
Gradient across the stenotic aortic valve (mm Hg) a	73.0 (60.0–85.0)	

Abbreviations: AR, aortic regurgitation; AS, aortic stenosis; BMI, body mass index; BSA, body surface area.

a Summary statistic is mean (standard deviation), all other continuous variables are summarized as median (interquartile range).

**Table 2:** Operative data of included patients

Operative variable	Included patients *n* = 31	
Cardiopulmonary bypass time, minutes	125.0 (90.0–160.0)	
Aortic cross-clamp time (min)	90.0 (60.0–110.0)	
*Valve replacement:*		
Single, *n* (%)	18	58.1%
Double, *n* (%)	13	41.9%
*Size of aortic prosthesis:*		
19 St. Jude reagent supra-annular	16	51.6%
21 St. Jude reagent supra-annular	14	45.2%
23 St. Jude reagent supra-annular	1	3.2%

Note: Continuous variables are summarized as median (interquartile range).

### Postoperative Outcomes

PPM was found in only one patient (3.2%), with mortality and reexploration being relatively uncommon as well (two patients experienced each complication, respectively), and that occurred in two cases of double-valve replacement. The duration of mechanical ventilation was short, as shown in Table 3 . Most importantly, the mean pressure gradient across the aortic valve at 3 years was 20 mm Hg, compared with 73 mm Hg measured before surgery. The functional orifice of the aortic valve also underwent notable change from 0.9 to 1.7 cm ^2^ at follow-up/postoperatively. There were no cerebrovascular events noted following surgery.

**Table 3:** Postoperative data of included patients

Postoperative variable		
ICU stay (d)	2.0 (2.0–3.0)	
Hospital stay (d)	5.0 (5.0–6.0)	
Duration of mechanical ventilation (h)	5.0 (5.0–6.0)	
Reexploration for bleeding, *n* (%)	3	9.7%
Postoperative mean gradient across the aortic valve (mm Hg)	19.00 (18.00–21.00)	
Postoperative aortic functional orifice (cm ^2^ )	1.7 (1.7–2.0)	
Patient-prostheses mismatch, *n* (%)	1	3.2%
Mortality, *n* (%)	2	6.5%
Temporary pacing, *n* (%)	2	6.5%
Duration of follow-up (mo)	38.0 (36.0–40.0)	
Mean gradient across the aortic valve at 3 years (mm Hg)	20.0 (18.0–20.0)	

Abbreviation: ICU, intensive care unit.

Note: Continuous data expressed as median (interquartile range).

First case mortality was in hospital eighth day in the ICU due to chest infection and respiratory failure. The second case mortality was in outpatient follow-up due to mediastinitis and repeated sternal dehiscence as the patient was diabetic and morbidly obese.

## Discussion

AVR is among the commonest interventions in cardiac surgical practice, used as treatment for valvular pathology of varying etiologies. This can be complicated by patients with small aortic annuli (of 21 mm or less), who may need to be implanted with a larger prosthesis. PPM can lead to increased left ventricular strain and impaired mass regression. 8 A meta-analysis of 34 observational studies demonstrated increased mortality in the PPM group of patients compared with those with no PPM. 9

Nick's procedure, or posterior aortotomy enlargement, attempts to ameliorate this by enlarging the aortic root through the noncoronary sinus, across the valve annulus, with a patch to support and extend the annulus.

This single-center, retrospective study examines the success of Nicks in a subset of patients with small aortic annulus, followed for 3 years. It is worth noting that the majority of aortic pathology was attributed to rheumatic heart disease, which may be in contrast to the previous literature, where the predominant etiology is calcification or degeneration.

Nick's procedure has reduced PPM substantially, with only one patient being noted to have PPM. Additionally, in-hospital mortality, ICU stay, and complication rates were low and in line with the literature. 10 Echocardiographic assessment at 3 years was favorable, depicting a significantly reduced mean pressure gradient across the aortic valve. These results are in accordance with similar retrospective studies done in Europe. Rammos et al studied the immediate and intermediate outcomes of 15 patients who needed Nick's procedure at a single center over a 10-year period. They reported an improvement in several key echocardiographic parameters, such as left ventricular end diastolic diameter, functional orifice of the aortic valve, and mean and peak pressure gradients. 10 Chowdhury et al 5 investigated immediate and intermediate outcomes of 115 patients who underwent Nick's procedure, as part of a single AVR or dual-valve replacement (with mitral valve placement) between 1997 and 2019. They reported no cases of PPM or pericardial patch aneurysm, a possible complication of the technique.

In a similar vein, Dhareshwar et al 6 investigated the profile and outcomes of 249 patients who required ARE. While the procedure itself was not associated with mortality, the patients who required enlargement were more likely to have comorbidities and be at higher risk of adverse outcomes. Balancing with the higher risk of adverse outcomes noted in the previous literature, the risks of ARE with Nick's procedure need to be weighed against its benefits on a case-by-case basis.

Peterson et al 11 collected data on 669 patients undergoing AVR and aortic annular enlargement from 1995 to 2005 and their finding were similar to us that ARE can be done safely in selected patients to avoid PPM. 11

## Limitations

These were the data from a single center in Egypt, and thus, the findings lack generalizability and applicability to a wider population. We are unable to draw conclusions on the differential outcomes between Nick's and other aortic enlargement techniques or patients without root enlargement due to the lack of a control group.

## Further Research

The literature regarding Nick's procedure is limited. Evaluation of long-term echocardiographic parameters combined with functional measures such as exercise tolerance would prove useful. Randomized controlled trials comparing Nick's with other aortic enlargement techniques are imperative to draw conclusions regarding relative efficacy.

## Conclusion

Our experience in a single center demonstrates Nick's procedure is a feasible, safe technique for use in patients requiring AVR with small aortic annulus. Further research is required regarding the effectiveness of Nick's in comparison to other techniques.
